# Bursts of regional cortical inhibition during smartphone use

**DOI:** 10.1016/j.isci.2026.115375

**Published:** 2026-03-16

**Authors:** Wenyu Wan, Arko Ghosh

**Affiliations:** 1 Cognitive Psychology Unit, Institute of Psychology, Leiden University, Wassenaarseweg 52, Leiden 2333 AK, the Netherlands; 2 Department of Psychology, University of Amsterdam, Nieuwe Achtergracht 129, Amsterdam 1001 NK, the Netherlands

**Keywords:** Neuroscience, Behavioral neuroscience, Cognitive neuroscience, Biomedical engineering, Psychology

## Abstract

Transient beta (β) bursts—brief neural events—are increasingly recognized for their role in motor and cognitive processes, yet their contribution to naturalistic behavior remains poorly understood. Smartphone behavior, involving continuous varied engagement, provides a rich real-world context to examine these dynamics. We recorded electroencephalography (EEG) as participants interacted with their smartphones using their right thumb for ∼80 min. β-bursts were detected across the scalp, with bilateral sensorimotor electrodes accumulating more bursts than other electrodes. Brain-wide burst probability decreased before touchscreen touches and increased afterward, with the strongest modulation over left sensorimotor cortex. Touches occasionally occurred during bursts, but at lower rates over left sensorimotor regions than elsewhere. Separating touchscreen intervals with versus without bursts revealed an exaggerated difference in sensorimotor cortex: intervals containing bursts were longer. These patterns suggest that continuous smartphone touches are modulated by brief, spatially localized transient bursts that may gate motor execution in sensorimotor networks.

## Highlights

• Smartphone interactions are interleaved with transient neural inhibition (β-bursts)
• Inhibitory activity is dominant over the sensorimotor cortex
• Behavior can escape inhibition, but the outputs are slower than when the inhibition is off
• Inhibitory transients may shape real-world behavioral outputs

## Graphical abstract

**Figure:**
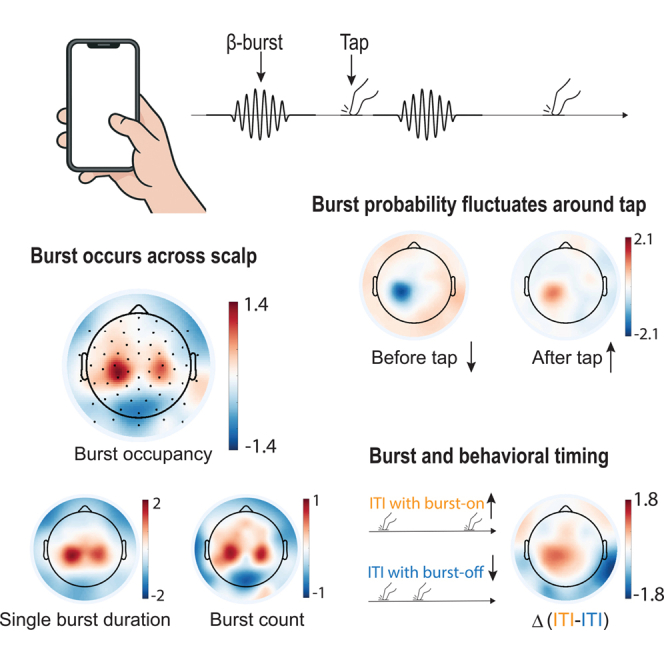

## Introduction

Rhythmic brain dynamics in the β-band (∼13–30 Hz) range are correlated with various cognitive processes, ranging from top-down sensory processing to the precise timing of motor outputs.^1^^,^^2^ There is an emerging idea that these oscillations occur transiently in bursts rather than being sustained over time.^3^^,^^4^ This perspective offers additional clues on the functional role of β-band activity, apart from offering a more accurate description of the underlying physiological signals.^1^^,^^3^ Both the conventional literature on beta activity and the emerging burst measurements show a reduction in sensorimotor beta activity surrounding movements followed by a rebound—although the bursts appear more spatially localized and are better predictors of behavior (especially β-burst timing) than the conventional measures (e.g., single trial β-amplitude).^5^^,^^6^^,^^7^^,^^8^ As the bursts appear across the cortex, one attractive idea is that they generally contribute to top-down neural processing by enabling transient and localized inhibition that supports goal-directed behavior.^3^ Moreover, these transients may enable inter-regional communication necessary for coordinating multiple cognitive processes, integrating information, and controlling motor outputs.^7^^,^^9^ The rich behaviors expressed in the real-world recruit multiple cognitive processes and involve complex event sequences. Capturing β-bursts in such naturalistic settings may help uncover their role in coordinating distributed computational processes across large-scale neural networks.

Smartphone behavior involves complex sensorimotor and cognitive processes that engage large and distributed neural populations. There are scattered efforts to leverage the quantifiable nature of such behavior to address the underlying cognitive process.^10^^,^^11^^,^^12^^,^^13^^,^^14^ The fluctuations in the amount of touchscreen interactions from 1 min to the next are reflected in the β-band power, such that higher use is associated with lower over the sensorimotor electrodes.^13^ Although the behavior involves a complex train of touchscreen interactions, time-locking the β-band power fluctuations to the touch reveals suppression around smartphone interactions across the scalp, predominantly over the sensorimotor electrodes.^12^ It remains unclear, however, whether these seemingly gradual modulations in average β-power reflect changes in the probability of β-bursts, rather than sustained oscillatory activity.

The idea that β-bursts offer brief and local inhibition to support top-down cognitive processes underlying goal-directed behavior raises a range of questions regarding their role in smartphone behavior. First, smartphone touchscreen interactions are multi-pronged processes involving sensory and motor processing. It is established that the location of β-bursts appears to be task specific.^3^ During smartphone behavior, do the bursts span several cortical areas? Second, β-bursts in the sensorimotor cortex are linked to motor inhibition.^15^ Given that touchscreen interactions are uninstructed, how does β-burst probability fluctuate around the touches—can spontaneous behavioral outputs emerge during ongoing somatosensory β-bursts? Third, smartphone behavior is continuous, with the user moving from one touchscreen interaction to the next with varying interval timing. Based on the idea that the bursts enable a “clear out” of working memory or recently evoked sensorimotor processes,^16^ does this process occur after each touchscreen interaction? Alternatively, are the transients more likely to occur for certain smartphone behaviors over others—for instance, do the behaviors characterized by long touchscreen interaction intervals show more bursts than those with shorter intervals?

Addressing such an array of questions rests on measuring and analyzing β-bursts during smartphone behavior. Here, we captured transient neural patterns in 54 individuals by using electroencephalography (EEG), while participants used their own smartphones in the laboratory for a period of ∼80 min. During the EEG recording, the participants were simply instructed to conduct smartphone activities that they commonly performed in the real world. We captured the spatial distribution of the bursts by recording across the scalp. We analyzed the bursts time-locked to the smartphone touchscreen events to reveal how the burst probability fluctuated surrounding the touch. Finally, we captured the nature of the smartphone behavior—in terms of touchscreen interaction dynamics—in the presence of bursts. Our findings reveal a temporal and spatial distribution of β-bursts aligned to the spontaneous smartphone behavioral dynamics.

## Results

### β-burst occupancy during smartphone use

**Figure 1:**
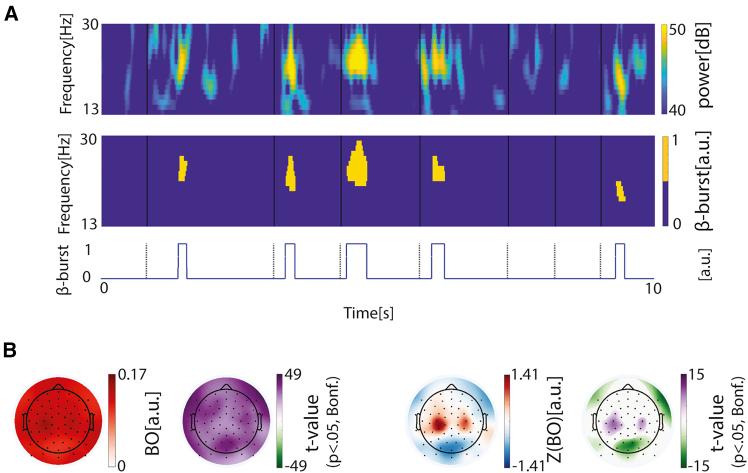
Spatial distribution of β-burst occupancy (in continuous EEG) during smartphone use (A) β-burst detection from continuous time-frequency activity. The top row shows 10 s of continuous beta-band (13–30 Hz) time-frequency activity. The middle row displays the corresponding binary β-burst detection based on power and duration thresholds. The bottom row shows a simplified binary representation, indicating the occurrence of β-bursts. (B) The left panel exhibits the grand-averaged BO (calculated as the total burst duration divided by recording time per electrode) across the population, along with t-statistics from one-sample *t* tests against zero, corrected for multiple comparisons using the Bonferroni method. The right panel has the same legend but displays results based on the z-normalized BO values.

We detected β-bursts while individuals interacted with their smartphone using only their right thumb. The duration of the bursts varied across participants, electrodes, and burst events. At the population level, the grand-averaged burst durations ranged from 151 to 181 ms across the scalp, based on per-electrode medians within participants (Figure S1). We estimated the β-burst occupancy (BO)—a proportion of recording time spent in bursts (sum of burst durations/total recording time). The bursts were observed across the scalp ranging from an occupancy from 0.10 to 0.15 at the population level (Figure 1). The bursts were common in the sampled population as indicated by the one-sample *t* tests against 0 (where a null value indicates an absence of bursts, Figure 1). To address the topology of the bursts, we z-normalized the BOs across the scalp. This transformation followed by a one-sample *t* test revealed a relatively high occupancy of burst over the bilateral sensorimotor cortex and a low occupancy at the posterior electrodes (Figure 1).

We further examined the duration of individual bursts and observed a pattern similar to that observed for BO, with bilateral sensorimotor electrodes showing longer burst durations than the average. We also found that burst counts were higher over bilateral sensorimotor electrodes compared with the overall mean (Figure S1).

In sum, the bilateral sensorimotor electrodes showed higher burst occupancy, driven by both longer individual burst durations and a greater number of burst events occurring in these regions.

### β-burst time-locked to the smartphone touchscreen touch

**Figure 2:**
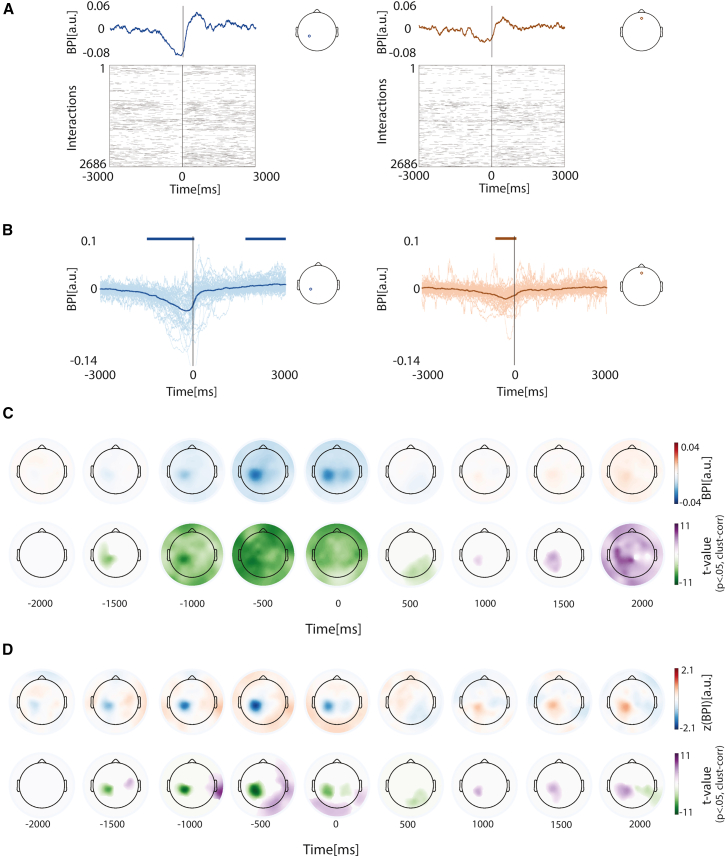
β-burst time-locked to smartphone touchscreen events (A) (Top) Baseline-corrected β-burst probability estimated from a raster of bursts time-locked to the touchscreen touch. Data from one representative participant and two electrodes (left: contralateral sensorimotor electrode, right: frontal electrode, location as indicated in inserts). (Bottom) Trial-by-trial binarized β-burst activity from −3 to +3 s relative to touchscreen touch. (B) Bold lines show the grand-averaged time-locked β-burst probability across participants for two electrodes (left: left sensorimotor; right: frontal, as shown in inserts). Thin lines represent individual participants. Top horizontal bars indicate time periods (>100 ms) with baseline-corrected β-burst probabilities significantly different from zero according to one-sample *t* test (*p* < 0.05, Bonferroni corrected). (C) (Top row) Scalp topography of grand-averaged time-locked BPI across participants at selected time points relative to interaction onset. (Bottom row) t-statistic from one-sample *t* test results comparing BPI to zero (*p* < 0.05, corrected for multiple comparisons using spatial-temporal cluster-based bootstrap approach; 1,000 resamples, α = 0.05). (D) (Top row) Same results with (C), but z-normalized across electrodes. (Bottom row) Same t-statistic results but based on the z-normalized BPIs.

Smartphone use consists of a complex sequence of touchscreen events (median inter-touch interval, ∼871 ms; interquartile range, ∼1,236 ms). We next addressed if and how the β-bursts are aligned to the isolated touchscreen events. To this end, we time-locked the bursts to the touchscreen event yielding a raster from which we determined the β-burst probability index (BPI) spanning −3 s–3 s from the touch (Figure 2A). This revealed a fluctuation of bursts in the immediate surroundings of the touch, such that the bursts were diminished ∼1,489 ms prior to the touch to rebound by ∼1,695 ms after the touch for the left somatosensory electrode across the population. A pre-touch suppression was also observed at frontal electrodes, where the burst probability diminished approximately ∼614 ms before the touch (Figure 2B, for an analysis spanning all electrodes, see below).

**Figure 3:**
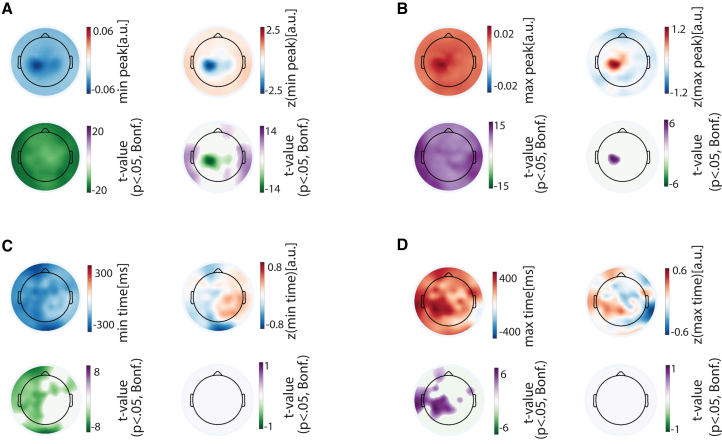
Magnitude and timing of β-burst suppression and rebound surrounding smartphone touchscreen events (A) (Left) The spatial distribution of the minimum (suppression) β-burst probability across the population. The adjacent topography shows the corresponding t-statistics from one-sample *t* tests against zero, masked at *p* < 0.05 with Bonferroni correction for multiple comparisons. (Right) The same results but based on z-normalized values across the scalp. (B) Same as in (A), but for the maximum (rebound) β-burst probability. (C) (Left) The latency of the minimum β-burst probability (suppression) and the corresponding t-statistics from one-sample *t* tests, masked at *p* < 0.05 (Bonferroni corrected). (Right) The same results but based on the z-normalized latency values. (D) Same as in (C), but for the latency of the maximum β-burst probability (rebound).

We addressed the time-locked fluctuations across the electrodes. This revealed a scalp-wide reduction in bursts starting at ∼1,100 ms prior to the touch to rebound by ∼2,000 ms (Figure 2C). We used z-transformations to reveal the differences between the electrodes. According to one-sample *t* tests, the left sensorimotor electrodes (contralateral to the movement) showed relatively strong burst suppression prior to the touch, whereas the same electrodes showed a rebound more than the rest of the electrodes after the touch. We quantified the fluctuations in the immediate temporal surrounding of the touch (−1s–1s) in terms of the extent and timing of the burst reduction and rebound (Figure 3). The minimum burst probabilities were confined to the left (contralateral) sensorimotor cortex. In terms of the maximum burst in this period, the sensorimotor electrodes showed the lowest values.

We leveraged the temporal resolution of β-burst in a subsequent analysis linking to the smartphone touchscreen event (Figure S2). The latency from the touch to burst onset was shorter over the left sensorimotor electrodes compared to other electrodes. The same regions also showed longer latencies from the burst offset to touch.

In sum, there were considerable fluctuations in β-bursts surrounding isolated smartphone touchscreen events. While there was brain-wide modulation in bursts surrounding the touch, the fluctuations were relatively exaggerated over the contralateral sensorimotor cortex—both in terms of burst suppression and a quick rebound.

### Smartphone touchscreen events surrounding β-bursts

The analysis of bursts time-locked to smartphone touchscreen events revealed fluctuations in burst probability, with a reduction prior to the touch. But these probabilities before baseline correction never really dropped to zero, suggesting that bursts may play a more flexible role in tuning behavior outputs. To further examine this possibility, we performed a series of analyses to characterize how bursts accompany behavioral output.

**Figure 4:**
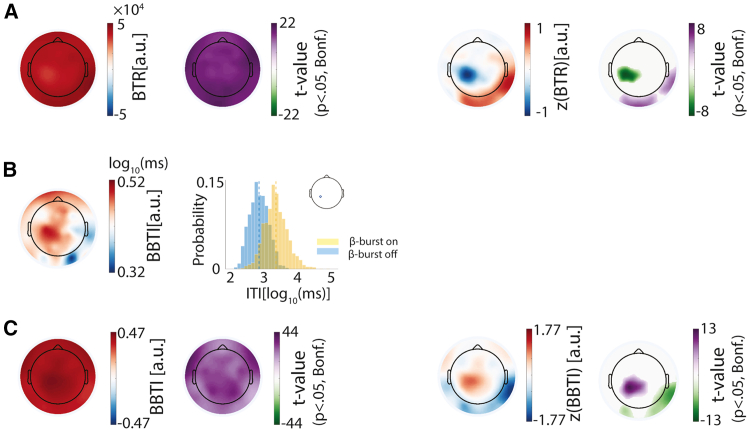
β-bursts modulate the likelihood and timing of smartphone behavior (A) Behavior probability when β-bursts occur across the scalp. (Left) The topography of grand-averaged interactions probability (z-transformed) during β-bursts. (Right) The t-statistic result from one-sample *t* tests against zero (masked by *p* < 0.05, Bonferroni corrected). (B) (Left) The temporal pattern difference (log10 scale) of behavior with and without β-bursts across the scalp from one participant (BBTI). (Right) The distribution of inter-interaction intervals with and without β-bursts for the sensorimotor electrode for this participant (as insert shown). (C) (Left) The topography of grand-averaged intervals distribution difference between with and without β-burst (BBTI) across participants and the t-statistic of one-sample *t* test against zero (masked by *p* < 0.05, Bonferroni corrected). (Right) The same results with left, but based on the z-normalized BBTIs.

First, we estimated the touch rate when the bursts were *ON* by dividing the number of touches occurring during a β-burst by the total β-burst duration. While the behavior did occur during bursts detected across the scalp, it was least probable for the bursts detected over the contralateral sensorimotor cortex and most probable for the burst detected over the occipital and temporal electrodes (Figure 4A). Second, we separated the smartphone touchscreen interaction intervals according to whether a burst occurred in the interval or not. Figure 4B (right) shows an example of the distribution of the inter-interaction intervals, with or without bursts, for a sensorimotor electrode from one participant. We quantified the difference between the interval duration distributions as the β-burst behavioral timing index (BBTI). Figure 4B (left) shows the topography of BBTI for the same participant. In general, the intervals were longer in the presence of bursts than without across the whole scalp in the population level (Figure 4C, left). When z-transformed, the contralateral sensorimotor electrodes showed relatively large gaps, whereas the right parietal electrode revealed the smallest gaps (Figure 4C, right).

In sum, these results consistently suggest that β-bursts do not strictly suppress movement but rather modulate both the likelihood and timing of behavior output by dynamically shifting between on and off states in a region-specific manner.

## Discussion

We found β-bursts across the scalp when participants engaged in smartphone behavior. Although smartphone behavior involves a range of cognitive processes—including sensorimotor processing, visual perception, and memory—there was a pronounced concentration of β-bursts and corresponding fluctuations over sensorimotor cortex. Smartphone behavior consists of a continuous train of touchscreen interactions. Although each touch is embedded in a train of behavioral outputs, we find a momentary reduction of burst probability surrounding an individual touch. Still, touches sometimes occur during the burst-ON periods, and bursts were more likely to be observed in behaviors with longer inter-touch intervals. These patterns suggest that, during real-world behavior, the precise spatiotemporal distribution of transient inhibitory neural processes supports a variety of parallel processes underlying smartphone behavior.

In general, there was a pronounced concentration of β-bursts in the sensorimotor cortex, and this was in keeping with prior observations where bilateral sensorimotor bursts are predominant, be it at rest or during behavior.^7^^,^^15^^,^^17^^,^^18^ The distinct circuitry of the sensorimotor cortex, including its dense pathways to and from subcortical structures that drive its activity (with subcortical regions such as the basal ganglia capable of generating numerous bursts), may partly explain the concentration of bursts.^19^^,^^20^^,^^21^ Alternatively, the observed β-burst concentration in the sensorimotor cortex may reflect the unique nature of peripheral somatosensory afferents,^19^^,^^22^ which continuously drive cortical processing during touchscreen interactions. Here, the β-bursts serve as a gating or filtering mechanism,^23^ continuously regulating cortical access to afferent sensory input from the sensory thalamus.^24^^,^^25^ Prior work has suggested that β-bursts of different durations may reflect distinct functional states, with longer bursts often linked to stronger or more sustained inhibitory processes in sensorimotor networks.^7^^,^^15^ We found that the bilateral sensorimotor electrodes showed longer individual burst durations than the other electrodes. This marks sensorimotor burst durations as an important feature for future work to explain touchscreen touches.

In the temporal window surrounding the individual touchscreen touches, we found that the burst probability across the scalp diminished and then rebounded, in keeping with what has been observed before using conventional measures of beta activity surrounding motor responses.^7^^,^^12^^,^^26^^,^^27^ This supports the idea that fluctuations in beta activity previously attributed to sustained changes occur at least partly through transient bursts in neural activity. The contralateral dominance of burst modulation further supports the interpretation that these effects are tightly linked to motor execution rather than reflecting global arousal or attentional fluctuations.

One widely held view is that β-band activity is associated with maintaining the current cognitive or motor status.^1^ From this perspective, the β-burst probability reduction before touches may reflect a release from the existing status, allowing a shift in cognitive activity or behavior. The β-burst probability increases after touches, which may reflect the re-establishment of stability. From one touch to the next, the timing of the bursts showed substantial variation, raising the possibility that β-bursts are timed according to a complex mixture of intrinsic—such as cortical excitability^28^—and extrinsic factors—such as kinematics of the thumb.^12^

Beta activity in the sensorimotor cortex has been mainly implicated in motor inhibition—as if the inhibition must be overcome to enable a movement.^15^ In our study, we found that the modulation of burst probability surrounding touchscreen touches appeared to support this inhibitory role. In particular, the sensorimotor bursts were the least “permissive” to the touchscreen touches, indicating that inhibition there may be the hardest to overcome during smartphone interaction. However, we did observe that touchscreen touches sometimes occurred during periods of β-bursts. One possibility is that, beyond motor inhibition and in parallel to it, β-bursts in the sensorimotor network also support functions such as sensory prediction, integration, and motor planning.^7^^,^^29^^,^^30^^,^^31^ The longer burst durations accompanying smartphone touches too may, therefore, reflect a composite of these overlapping functional roles. Another possibility, that we deem plausible, is that smartphone use engages a dynamic competition between internal and external processing, with β-bursts modulating this balance^25^: when β-bursts are on, internal processing dominates and external processing is suppressed, whereas when β-bursts are off, external inputs prevail and internal processing is inhibited. Another possibility is that β-bursts do not strictly serve as inhibitory gates to suppress movement but rather modulate motor behavior by slowing or delaying its execution. Supporting this possibility, the smartphone intervals between touchscreen touches with bursts in the sensorimotor cortex were longer than the intervals without the bursts. Together, these findings argue against a strictly all-or-none motor inhibitory role of β-bursts and instead support a modulatory framework in which bursts bias the timing and likelihood of action.

Moreover, the behaviors with longer intervals may be supported by more diverse cognitive processes than those with the shorter intervals. The bursts may be necessary to coordinate these diverse processes spanning well beyond the sensorimotor cortex.^9^^,^^23^^,^^30^^,^^32^^,^^33^^,^^34^^,^^35^^,^^36^ In our observations, the gap between the interval distributions with vs. without burst spanned across the scalp, albeit the gap was the most prominent in the sensorimotor cortex. This is consistent with the idea that the inhibitory transients reflected in the β-bursts are used across the scalp for coordinating neural processes.^3^ There is a methodological consideration underlying the pattern linking the bursts to longer intervals. Essentially, the longer the interval, the higher the chance of encountering a burst simply due to the longer sampling period. Still, this aspect alone cannot explain the resulting topology. While the bursts were distributed across the scalp, the observed pattern was particularly pronounced over the sensorimotor cortex.

Distinguishing among the different interpretations of how β-bursts contribute to the continuous behavioral outputs expressed in the real world will require future studies that can experimentally manipulate burst timing or selectively interfere with specific cognitive processes during smartphone interaction. At present, our data demonstrate that the functional relationship between β-bursts and behavior is spatially heterogeneous and temporally complex, likely depending on the precise timing, current state of the network, and cortical location of the bursts.

In conclusion, brain-wide β-bursts accompany smartphone interactions. β-bursts are typically studied in laboratory-designed speeded tasks with a clear onset and offset. They are a signal of interest in clinical research focused on movement disorders and used, for instance, to explain parkinsonian symptoms.^20^^,^^37^^,^^38^^,^^39^^,^^40^^,^^41^ The continuous behavioral outputs captured on the smartphone offer a fresh perspective on these inhibitory transients. β-bursts have been implicated in a range of processes—from clearing working memory (WM) to action inhibition.^3^ The inseparable mixture of motor and cognitive processes in continuous and spontaneous smartphone behavior offers a fresh avenue to study β-bursts. Their distribution in a ubiquitous behavior may not only help address basic questions on the shared neural mechanisms spanning different cortical areas but also help yield a potential marker for abnormal brain functions.

### Limitations of the study

We deliberately permitted participants to use their own smartphone (with limited constraints) to study the role of β-bursts in continuous behavior—capturing a key feature of real-world behavior. Intuitively, this approach involves substantial intra-individual differences driven by various intrinsic and extrinsic factors. Intrinsic factors are, for instance, the level of alertness or attention, and extrinsic factors are, for instance, the content displayed on the screen or the type of app. Similarly, there may be substantial inter-individual differences from sensorimotor skills to how they use them in daily living. Despite these various factors, we discovered patterns that were consistently present across apps and individuals. This suggests that these patterns of β-burst dynamics reflect fundamental aspects of self-paced touchscreen interactions. In addition, factors such as gender, age, and handedness may influence β-burst dynamics during naturalistic smartphone use, and future work could explore these effects in a more targeted manner.

Our behavioral observations were focused on the timing of smartphone touchscreen interactions. This offers low-dimensional perspective of the rich smartphone behaviors, and future studies using additional sensors may offer a more complete behavioral description. For instance, our work using a kinematic sensor demonstrates that there is motor activity in between the touchscreen touches.^12^

Another consequence of the focus on touchscreen interactions is that some of our results are interpreted with a sensorimotor focus. There is emerging evidence for the role of various processes underlying the touchscreen interactions: from sensorimotor to executive processes.^12^^,^^13^^,^^42^ For instance, a rise in frontal theta activity is observed during smartphone use, indicative of heightened cognitive load.^13^ Our observations reveal strong time-locked modulations of β-bursts over the left sensorimotor cortex (contralateral to the right hand in use). However, we also observed scalp-wide modulations, and these modulations are likely to reflect cognitive processes in addition to sensorimotor control of the thumb. Future research to manipulate the bursts can help clarify whether they causally influence the behavior.^43^

## Resource availability

### Lead contact

Requests for further information and resources should be directed to and will be fulfilled by the lead contact, Arko Ghosh (a.ghosh@fsw.leidenuniv.nl).

### Materials availability

This study did not generate new materials.

### Data and code availability

• The processed data have been deposited at https://osf.io/k27n5/overview.
• Code for preprocessing analysis has been deposited at https://github.com/CODELABCODELIB/JID_ERP_Smartphone_2024; code for β-burst identification and subsequent statistical analyses are shared at https://github.com/CODELABCODELIB/BetaburstInhibition_2025.
• Any additional information required to reanalyze the data reported in this paper is available from the lead contact upon request.

## Acknowledgments

This study was funded by 10.13039/100007214Velux Stiftung (grant no. 1283, awarded to A.G. with K. Richard Ridderinkhof as co-applicant) and CSC (no. 202004910349, awarded to W.W.). We thank K. Richard Ridderinkhof for his substantial help with editing and revising the manuscript. We acknowledge the assistance of Beste Yavuz, Lysanne Groenewegen, Barbora Michalidesová, and Lorenzo Van Hoorde in EEG data collection. We are also grateful to all participants for their contribution to this study.

## Author contributions

A.G. conceived the study. A.G. and W.W. designed the study. W.W. acquired and performed EEG preprocessing and conducted the analyses with the aid of A.G. W.W. drafted the manuscript with the aid of A.G. A.G. and W.W. edited and revised the manuscript.

## Declaration of interests

A.G. is a co-founder and chairman of QuantActions AG and is an advisor for Axite B.V.

## Declaration of generative AI and AI-assisted technologies in the writing process

During the preparation of this work, the authors used Claude in order to improve manuscript clarity and readability. After using this tool or service, the authors reviewed and edited the content as needed and take full responsibility for the content of the publication.

## STAR★Methods

### Key resources table

**Table:** 

REAGENT or RESOURCE	SOURCE	IDENTIFIER
**Software and algorithms**		

MATLAB	MathWorks	RRID:SCR_001622
EEGlab 2024	SCCN/UCSD	RRID:SCR_007292
LIMO EEG toolbox	Pernet et al.^44^	RRID:SCR_009592
Movement model	Kock et al.^12^	https://github.com/CODELABLEIDEN/Non_goal_directed_smartphone_2022
Data preprocessing	Wan et al.^45^	https://github.com/CODELABCODELIB/JID_ERP_Smartphone_2024
Beta burst identification and statistical analysis	This paper	https://github.com/CODELABCODELIB/BetaburstInhibition_2025

### Experimental model and study participant details

#### Participants

We recruited participants via the agestudy.nl research platform.^46^ Inclusion criteria were: (1) healthy participants aged over 16 years; (2) ownership of an unshared android operating smartphone. Exclusion criteria included: (1) dysfunctional hand use; (2) self-reported sensitive skin; (3) need for mobility assistance; (4) recent changed in health status. The final sample consisted of 64 participants (age range: 20-81 years). 52 participants completed the handedness questionnaire, and 45 of them were classified as right-handed. This set of participants has been previously reported in Wan and Ghosh.^45^ All participants reported that they had no history of neurological or psychiatric disorders. Four participants were excluded because of the updated self-reported unhealthy status soon after the measurement session. Two participants were excluded because their smartphone data were unavailable. Four participants were excluded due to the poor clock alignment across the different instrumentation (see details below). After exclusions, a final sample of 54 participants was retained for analysis. All participants provided informed consent. This study was approved by the Ethics Committee of Psychology at Leiden University (ERB-reference number: 2020-02-14-Ghosh, dr. A.-V2-2044).

### Method details

#### Smartphone data collection

Smartphone behavior was recorded using the TapCounter app (QuantActions AG, Switzerland) at background, which was installed on participant’s smartphone.^10^ The app continuously logged the timestamps of all smartphone touchscreen events with millisecond precision in UTC, along with the label of the app in use. Participants were seated in a Faraday room and used their smartphone freely, interacting with the touchscreen using only their right thumb (as instructed) during ∼80 min of EEG recording. Every 10 minutes, after a brief break, participants were asked to switch to one of their four most frequently used apps from the past month (e.g. What’s app, Instagram, Google chrome); apps involving streaming audio or video content were explicitly excluded. Two experimenters monitored the session via livestream from an adjacent control room and reminded participants to use their right thumb only when needed.

#### Movement sensor recording and clock alignment

The timestamps recorded on the smartphone used a different internal clock than the laboratory EEG system. To synchronize these devices without invasive smartphone manipulation, we leveraged an existing approach using an additional movement sensor.^12^ Details of this procedure are provided below.

Movement data were recorded using a movement sensor (Flex Sensor, 112 mm, Digi-Key, Thief River Falls). The sensor was attached to the dorsum of the participant’s right thumb. This setup allowed participants to interact with smartphone freely. The analogue signals from the sensor were sampled at 1 kHz using the Polybox (Brain Products GmbH, Gilching, Germany), and were stored in the EEG dataset file, sharing the same clock as the EEG recordings.

Movement signals were processed following the procedure described by Kock et al.^12^ In brief, the raw signals were filtered between 1 and 10 Hz. The continuous signal was then epoched ranging from -3 to 3 s relative to the onset of smartphone touchscreen touches. Movement signals were averaged across touch events for each participant.

Since the smartphone clock may differ from the laboratory clock, we corrected for clock differences following the procedure described in previous studies.^12^^,^^45^ Briefly, a pre-trained model was used to estimate the screen touch times based on the movement signal. This model, developed by Kock et al.,^12^ employed a global bidirectional LSTM regression framework trained to predict force-sensor signals from movement-sensor inputs, with peaks in the predicted force used to infer touchscreen interaction times. Then, the time delay between the predicted touches and the smartphone-recorded touches was corrected. For participants who have more than one recording session (say induced by a bathroom break or impedance check), the predicted touch time may slightly differ across sessions because of kinematic differences between sessions. We then aligned the movement waveforms using *alignsignals* from Matlab’s Signal processing toolbox (MathWorks, Natick, MA, USA).

#### EEG recording and preprocessing

EEG data was collected by using 64 channels actiCap Snap cap (62 scalp electrodes, 2 ocular electrodes) with customized equidistant layout (Brain Products GmbH, Gilching, Germany). The EEG signals were gathered referenced to the vertex and amplified with BrainAmp amplifier (Brain Products GmbH, Gilching, Germany). The sampling rate of recorded and digitalized signals was 1 kHz. Participants were asked to arrive for the measurement with washed hair and scalp. The skin at the contact sites were further degreased by using alcohol swabs. Supervisc gel (Easycap GmbH, Herrsching, Germany) was applied to obtain an electrical contact between the skin and electrode, and targeted an impedance under 10 kΩ for each electrode. The entire EEG measurement lasted approximately 1.5 hours in total.

We used the EEGLAB toolbox^47^ and custom scripts (shared on GitHub) on MATLAB 2023b (MATLAB, Mathworks, Natick) to pre-process the EEG data. EEG data was high pass filtered at 0.5 Hz. The behaviourally inactive data segments – determined based on touchscreen touches – were removed if both pre- and post-touchscreen event intervals were longer than 30 s. Adaptive mixed independent component analysis was used to decompose independent component.^48^^,^^49^ Based on AMICA results, we rejected artifact components with over than 90% likelihood originating from artifacts like eyeblinks, muscle, etc., as evaluated by ICLabel toolbox.^50^ EEG data was further band-pass filtered with 1-45 Hz. We rejected bad channels with the correlation less than 0.85 with neighbouring channels by *clean_channels*. Rejected channels were interpolated using the spherical interpolation method by *pop_interp*.

#### β-burst detection

We used the β-burst detection procedure as described in Shin et al.^29^ and Wessel.^15^ Briefly, a complex Morlet wavelet approach was used to decompose the time-frequency (TF) power on the continuous EEG data spanning 13 to 30 Hz (Figure 1A). An individual β-burst was detected when power was greater than 6× the median of power spanning the whole recording period. β-burst durations less than 2 cycles for each frequency were further excluded. We then obtained a binary β-burst TF matrix. From this binary burst TF matrix, we further derived a binary β-burst time series.

### Quantification and statistical analysis

#### β-burst occupancy (BO)

We calculated the β-burst occupancy based on the continuous binary β-burst time series data, defined as the total burst duration divided by the whole recording duration. The calculation was performed for each participant across all electrodes. To study the differences between the electrodes, the β-burst occupancy was z-normalized across the electrodes. For population level statistics, we pooled either the BBO or the z-normalised values and subsequently conducted a one-sample t-test against zero using limo_eeg toolbox,^44^ followed by Bonferroni correction for multiple comparisons.

#### Time locked β-burst probability index (BPI)

We calculated the BPI for each participant and electrode by epoching the continuous binary β-burst time series from -3 to 3 s relative to the onset of smartphone touch, averaging across trials, and correcting baseline by subtracting the median value across the time. BPIs were normalized across electrodes to emphasize the difference in spatial distribution for each time point. Population-level analyses were performed by pooling BPIs or the z-normalised values across participants, and conducting one-sample t-tests against zero, with multiple comparisons corrected via spatial-temporal clustering (1000 bootstraps, α = 0.05). Topographies suggested variability in the timing and extent of β-burst suppression across electrodes. To capture this variability across electrodes, we identified the minimum value of the BPI within the –1 to 1 s window (relative to touch onset) for each electrode, along with the corresponding time point. We also normalized those values across the scalp to emphasize spatial variation. We pooled all raw and z-transformed values of suppression timing and magnitude across participants and performed one-sample t-tests against zero, with Bonferroni correction for multiple comparisons. A similar analysis was conducted to characterize β-burst rebound by identifying the maximum value and corresponding time point within the -1 to 1 s window relative to touch onset. One-sample t-tests were performed on the pooled raw and z-transformed values of rebound timing and magnitude, followed by Bonferroni correction.

#### Intra-burst touch rate (BTR)

From the time-locked β-burst probability index result, we observed that touchscreen touches sometimes occurred during periods of β-bursts. To quantify how extent β-bursts co-occur with touches, we calculated the burst-on touch rate by dividing the number of touches that occurred during β-bursts by the total β-burst duration. The BTRs were then z-transformed across the scalp to emphasize spatial difference. One-sample t-test against zero, followed by Bonferroni multiple comparison correction, was conducted on the pooled BTRs and z-transformed values.

#### β-burst behavior timing index (BBTI)

To examine how β-bursts relate to behavioral timing, we separated touchscreen intervals with and without the bursts and compared the timing of behavior between these two conditions. In brief, we separated the binary β-burst time series based on inter-touch intervals. Each data segment was categorized as with β-burst group if any burst occurred within the interval, or categorized as without β-burst group otherwise. We then calculated the difference in the median behavior timing (log_10_ scale) between those two groups for each electrode as BBTI. The BBTIs were then normalized across electrodes. We performed one-sample t-tests against zero on the pooled BBTIs and z-transformed values across the population, followed by Bonferroni multiple comparison correction.
