# Supplementary material for: Bursts of regional cortical inhibition during smartphone use
Source: iScience. 2026 Mar 16;29(4):115375. doi: 10.1016/j.isci.2026.115375 (PMC13068570; doi:10.1016/j.isci.2026.115375)
Supplement: Document S1. Figures S1 and S2 and supplemental methods [file mmc1.pdf]

iScience, Volume *29*

Supplemental information

Bursts of regional cortical inhibition during smartphone use

Wenyu Wan and Arko Ghosh

Supplementary methods

β-burst duration (BD)

We calculated β-burst duration (BD) from the continuous binary β-burst time-series data. For each participant and each electrode, the median duration of all detected bursts was obtained, and these values were then averaged at the population level for each electrode. To examine differences across scalp, BD were z-normalized across electrodes. For population-level statistics, we pooled either the raw BD or the z-normalized values and performed one-sample t-tests against zero using the LIMO EEG toolbox[1], followed by Bonferroni correction for multiple comparisons.

β-burst counts (BC)

β-burst counts (BC) were computed for each participant and each electrode. These values were then averaged at the population level for each electrode. To examine differences across electrodes, BC were z-normalized across electrodes. For population-level statistics, we pooled either the raw BC or the z-normalized values and performed one-sample *t*-tests against zero using the LIMO EEG toolbox[1], followed by Bonferroni correction for multiple comparisons.

Burst-tap lag

To examine the relationship between bursts and upcoming touchscreen taps, we calculated, for each participant and each electrode, the median interval between the burst end time and the subsequent touchscreen tap. These values were then averaged at the population level. To assess differences across electrode, burst–tap lag values were then z-normalized across electrodes. For population-level statistics, we conducted one-sample *t*-tests against zero on both the raw and z-transformed values, followed by Bonferroni correction for multiple comparisons.

Tap-burst lag

Using the same procedure with burst-tap lag, we calculated the median interval between each touchscreen tap and the onset of the subsequent first burst to examine the relationship between taps and upcoming bursts.

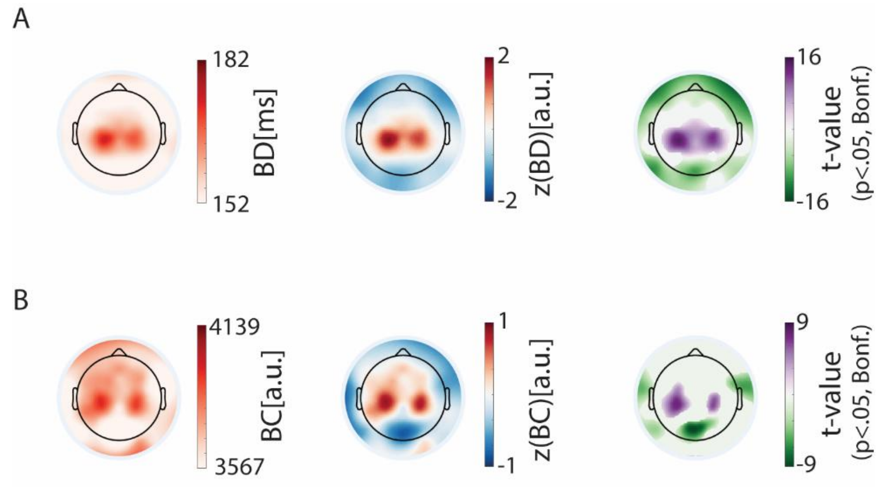

Supplementary Figure 1. Spatial distribution of β-bursts duration (BD) and counts (BC) during smartphone use.

(A) The left panel exhibits the grand-averaged β-bursts duration (BD) across the population. The middle panel has the same legend but displays results based on the z-normalized BO values and right panel shows t-statistics from one-sample t-tests against zero, corrected for multiple comparisons using the Bonferroni method.

(B) Same legend with (A) but for β-bursts counts (BC).

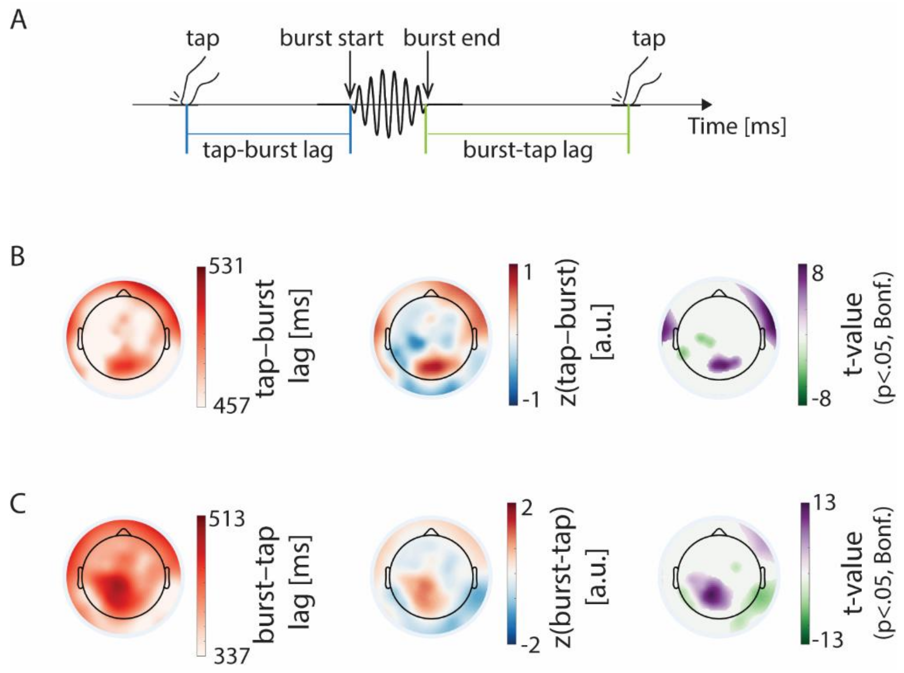

Supplementary Figure 2. Spatial distribution of tap-burst and burst-tap latencies during smartphone use.

(A) The sketch of calculating the tap-burst lag and burst-tap lag. The temporal gap between the tap and the subsequent burst onset is defined as the tap-burst lag (blue line) while the period between the burst offset and the subsequent tap is defined as the burst-tap lag (green line).

(B) The left panel exhibits the grand-averaged β-bursts tap-burst lag across the population, The middle panel has the same legend but displays results based on the z-normalized tap-burst lag values. The right panel shows t-statistics from one-sample t-tests against zero for the z-normalized tap-burst lag values, corrected for multiple comparisons using the Bonferroni method.

(C) Same as (B), but for the burst-tap lag.

References:

1. Pernet, C.R., Chauveau, N., Gaspar, C., and Rousselet, G.A. (2011). LIMO EEG: a toolbox for hierarchical LInear MOdeling of ElectroEncephaloGraphic data. Computational intelligence and neuroscience *2011*, 831409.
